# The clinical effectiveness of fused image of single-photon emission CT and facial CT for the evaluation of degenerative change of mandibular condylar head

**DOI:** 10.1186/s40902-023-00399-1

**Published:** 2023-09-27

**Authors:** Seung-Hwan Jeon, Seung-Weon Lim, Ki-Hyun Jung, Jae-Yun Jeon, Sang-Yoon Kim, Ji-Young Kim, Yoon-Young Choi, Kyung-Gyun Hwang

**Affiliations:** 1 Division of Oral and Maxillofacial Surgery, Department of Dentistry, College of Medicine, Hanyang University, 222-1 Wangshimri-Ro, Seongdong-Ku, Seoul, 04763 Korea; 2 Division of Orthodontics, Department of Dentistry, College of Medicine, Hanyang University, Seoul, Korea; 3 Smart Oral Health Care Research Center, Hanyang University, Seoul, Korea; 4 Former Resident, Department of Oral and Maxillofacial Surgery, Massachusetts General Hospital, Harvard Medical School, Boston, USA; 5 Private Practice, Vienna, VA Austria; 6 Department of Nuclear Medicine, College of Medicine, Hanyang University, Seoul, Korea

**Keywords:** Osteoarthritis, Bone SPECT, CT, Temporomandibular joint, Condyle resorption

## Abstract

**Background:**

The primary objective of this study was to assess the clinical effectiveness of fused images obtained from single-photon emission computed tomography (SPECT) and facial computed tomography (CT) for evaluating degenerative changes in the mandibular condylar head. This assessment was accomplished by comparing the Technetium-99 m methylene diphosphonate (^99m^Tc-MDP) uptake ratio with the results of clinical and radiographic findings.

**Methods:**

The study included 17 patients (3 males and 14 females) with suspected osteoarthritis of the mandibular condyle, totaling 34 temporomandibular joints (TMJs). Based on clinical and radiographic examinations, the TMJs were categorized into four groups: normal (group N), internal derangement (group ID), osteoarthritis (group OA), and osteoarthritis sequelae (group OA_seq_). For each patient, bone SPECT and facial CT scans were registered and reconstructed to create fused SPECT/CT images. The ^99m^Tc-MDP uptake levels in the TMJs were statistically compared among the four groups.

**Results:**

The ^99m^Tc-MDP uptake ratio showed a gradual increase in the order of the following: group N, group OA_seq_, group ID, and group OA. There was a significant difference observed among groups (*p* = 0.003), mainly driven by the disparity between group OA and both group N (*p* < 0.001) and group OA_seq_ (*p* = 0.048).

**Conclusion:**

Fused SPECT/CT image can be an effective tool for evaluating degenerative changes in the mandibular condylar head. The technique demonstrated the ability to differentiate between normal TMJs and those with internal derangement, osteoarthritis, or osteoarthritis sequelae. This approach holds promise as a valuable method in clinical assessments of TMJ degeneration.

**Supplementary Information:**

The online version contains supplementary material available at 10.1186/s40902-023-00399-1.

## Background

Osteoarthritis with degenerative change of mandibular condylar head is a group of overlapping distinct disease which may have different etiologies but with similar biologic, morphologic, and clinical outcomes [1]. Osteoarthritis is progressed by mechanical stress on the joint and low-grade inflammatory processes [2]. The disease not only affects the articular cartilage but also involves the entire joint, including the subchondral bone, ligaments, capsule, synovial membrane, and periarticular muscles. The articular cartilage degenerates with fibrillation, fissures, and ulceration, and the full-thickness joint surface is ultimately lost. Most common symptoms are joint pain and decreased range of motion [3].

Diagnosis of the osteoarthritis on temporomandibular joint (TMJ) has mainly performed on clinical findings using the research diagnostic criteria for temporomandibular disorder (RDC/TMD) chart and plain radiography. Even though the plain radiography such as panoramic view and transcranial radiography are useful tool to diagnose osteoarthritis, it has limitation in detecting lesions of early stages, since lesions can be observed when there are 30 to 50% of changes in bone mineral mass [4, 5]. The computed tomography (CT) on TMJ can improve detection accuracy of bone morphological changes and anatomical location [6], but changes in blood flow and bone metabolism around TMJ were difficult to detect. Therefore, a bone scan has been performed to distinguish joint status change even when the change in bone minerals is only 3 to 5% [7, 8], the bone scan provides extensive presence of bone activity change around the jaw joint, but it is difficult to accurately diagnose acute changes or conditions of bony surface of TMJ. In the field of nuclear medicine, bone scintigraphy and single-photon emission computed tomography (SPECT) using Technetium-99 m methylene diphosphonate (^99m^Tc-MDP) have been applied to identify the status of the local metabolic alteration in bone and joint. This was considered a useful technique which can upgrade the sensitivity of bone scan and used for the assessment of the transplanted bone for degenerative disease, avascular necrosis after surgery [9, 10]. However, SPECT also have limitation in terms of lower possibility of identifying exact anatomical location related with the pathogenesis and inflammation [11]. Therefore, the fused image of the functional SPECT image and morphological CT image has been expected to be an effective radiographic tool to characterize the bone pathology [12]. Through the fused image, both anatomical location and status of metabolic alteration of bone lesion were expected to be identified, which would get over the limitations of existed imaging [13–15].

However, to the best of our knowledge, there were few studies on clinical usefulness of the fused image of bone SPECT and facial CT in the diagnosis of osteoarthritis of the TMJ. Therefore, the objective of this retrospective study was to evaluate the clinical effectiveness of fused image of SPECT/CT for the evaluation of degenerative change of mandibular condylar head by comparing the ^99m^Tc-MDP uptake ratio obtained in the fused image with the results of clinical and radiographic findings. The null hypothesis of this study was that the fused image of SPECT/CT would not be effective for the evaluation of degenerative change of mandibular condylar head.

## Methods

### Subjects

The fused SPECT/CT image of 34 mandibular condyles of the 17 patients (3 males and 14 females) who visited the Department of Oral and Maxillofacial Surgery, Hanyang University Hospital, was retrospectively collected. All the 17 patients were suspected to have osteoarthritis of mandibular condyle on plain radiography and clinical examination; therefore, facial CT and bone SPECT were taken for further evaluation. The age distribution ranged from 14 to 66 years, with a mean age of 34.3 years. The protocol for this study was reviewed and approved by the Institutional Review Board (IRB) of the Hanyang University Hospital (HYUH 2012–11-009–012). The requirement for patient consent was waived by the IRB committee.

### Diagnostic criteria and image acquisition

Primary diagnosis was based on DC/TMD Axis I (clinical physical examination), the clinical findings (palpation, subjective pain report, any coarse crepitus sound, and pain during movement), and bony change on plain radiography (erosion, sclerosis of cortical bone, osteophyte formation).

And then facial CT and bone SPECT of the patients were acquired. Facial CT (Sensation 16, Siemens, Berlin, Germany) was taken with exposure parameters at 120 kV, tube current 80 mA s/slice, and 1-mm slice thickness. Facial CT scan time was 10 s covering 17 cm. The estimated CT radiation dose was 6.5 mGy to each patient.

The bone SPECT was acquired 4 h after the intravenous administration of ^99m^Tc-MDP (740 ~ 1110 MBq). The SPECT was taken on a dual head gamma camera (ECAM, Siemens Medical System, Chicago, IL, USA) using a low-energy, ultrahigh resolution collimator in a 128 × 128 matrix in continuous mode for 64 views per detector over 180° for 20 s per view. The bone SPECT data were reconstructed using OncoFlash (Siemens, Erlangen, Germany).

### Group of the patients

According to the clinical and radiographic examinations of plain radiography, 34 TMJs of the 17 patients were divided into 4 groups. Thirty-four condyles were identified as anonymized patient number (from 1 to 17), and the right and left condyles were indicated as R and L, respectively (Supplemental Table).

- Normal group (group N) included the TMJs defined as no symptom except occasional clicking sound and normal feature on radiographic exam.
- Internal derangement group (group ID) was defined as an abnormal relationship between the articular disc and the mandibular condyle, articular fossa, and the articular eminence. These group represented the symptom of joint noise during normal function, mouth opening limitations, temporary joint locking, and pain without any bony change on plain radiography. These TMJs belonged to the group II of DC/TMD Axis I.
- Group osteoarthritis (group OA) and group osteoarthritis sequelae (group OA_seq_) were belong to the group III of DC/TMD Axis I. In these groups, bony change such as bone erosion, cortical thinning, sclerosis, osteophyte, and irregularity on condylar head was observed on radiographic exam. Group OA showed the symptom of arthralgia on TMJ, and group OA_seq_ showed no clinical symptom except crepitus.

Single rater, who was profession of the Department of Oral and Maxillofacial Surgery, assessed the TMJs and performed grouping, and another rater performed the grouping separately. The result of grouping coincided with each other.

### Image analysis of fused image of bone SPECT and facial CT

**Fig. 1:**
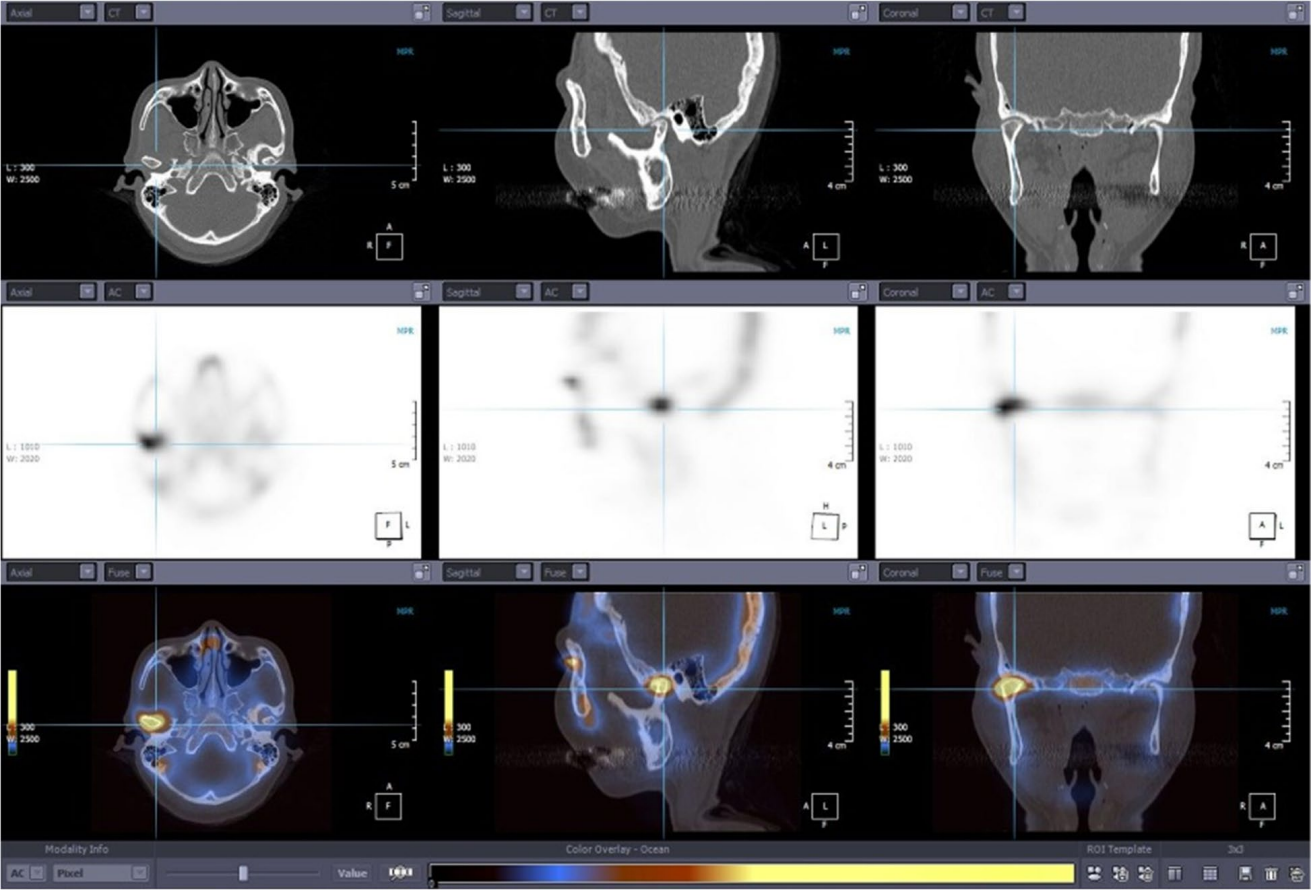
Facial CT, Bone SPECT and co-registered fused image using Xelis program in the transverse, coronal, and sagittal planes, showing focal hyperactivity of the radiopharmaceutical in the left TMJ

SPECT data and CT data for each patient were transferred and co-registered to yield fused SPECT/CT image in axial, coronal, and sagittal plan using 3D software (Xelis, INFINITT Healthcare, Seoul, Korea) (Fig. 1).

**Fig. 2:**
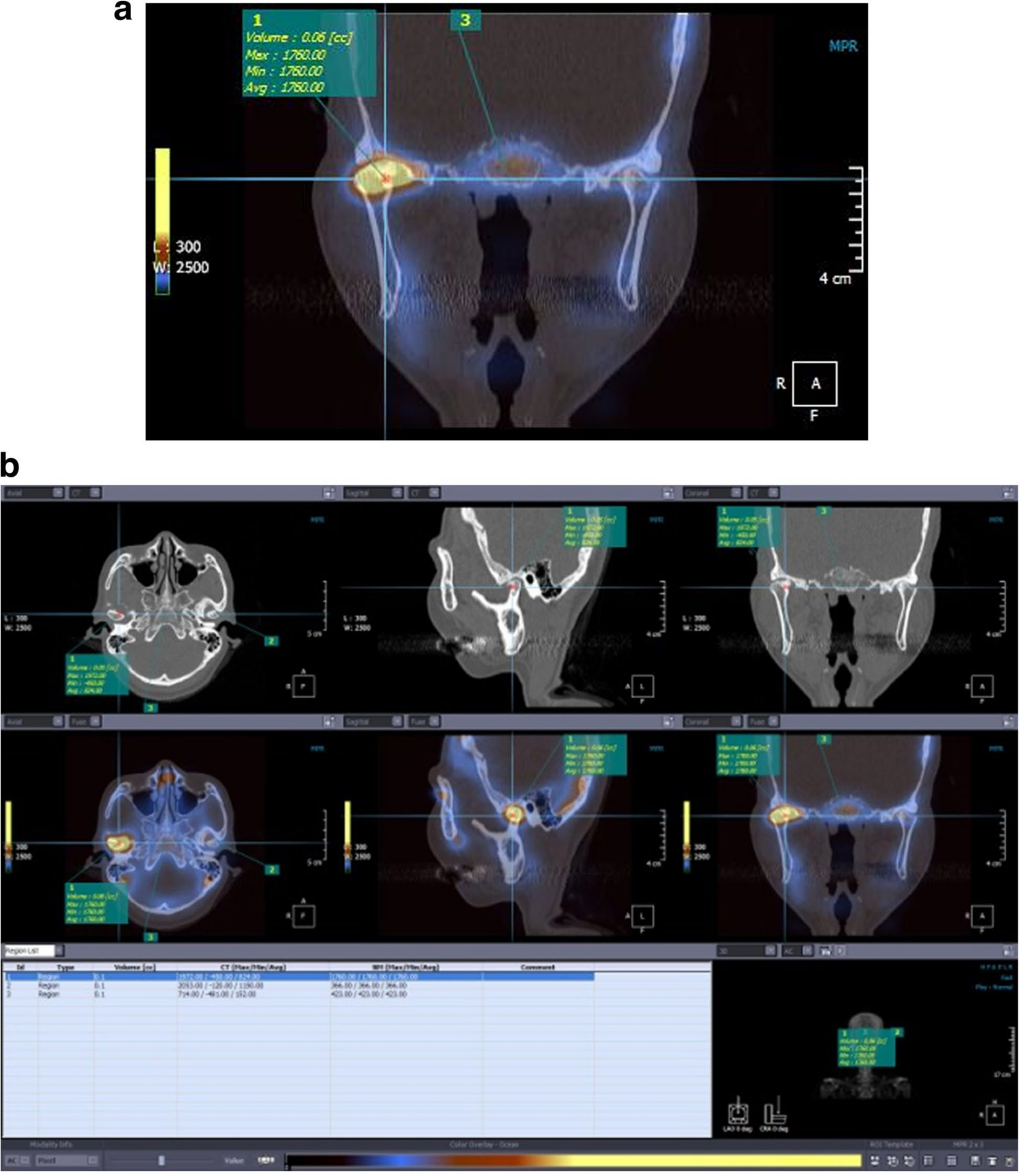
Region of interest (ROI) drawn over the right condyle to calculate the maximum, minimum, and average radiotracer uptake count on bone SPECT and facial CT-fused image. **a** Radiotracer count was calculated by ROI (3.0 × 3.0 × 3.0 pixel) on coronal image.** b** Radiotracer count was calculated by ROI (3.0 × 3.0 × 3.0 pixel) on 3 planes (axial, sagittal, coronal view). Right, left condyle, and clivus also calculated for the ratio in each subject

To quantitate the ^99m^Tc-MDP uptake level of the TMJ, a sphere shape region of interest (ROI) (3.0 × 3.0 × 3.0 pixel) was designated in the highest point for evaluation and the counts in both the condyles and clivus measured (Fig. 2). For the bilateral condyle regions, the uptake ratio was obtained using the clivus as a background measurement. The average values were used to calculate uptake ratio (TMJ uptake ratio = average count at condyle/average count at clivus). Single rater experienced nuclear medicine clinicians, evaluated the uptake level of ^99m^Tc-MDP of TMJ through visual analysis, and after 2 weeks, same and another rater repeated the evaluation. Both inter- and intra-examiner reliability showed excellent agreement; the intraclass correlation coefficient value was 0.892 and 0.922, respectively.

### Statistical analysis

Statistical analyses were performed using the SAS software, version 9.2 (SAS Institute Inc., Cary, USA). Kruskal-Wallis test, and further multiple comparisons using Tukey test for the post hoc analysis, was conducted to evaluate differences between groups. Value of *p* < 0.05 was considered to be significant.

## Results

**Fig. 3:**
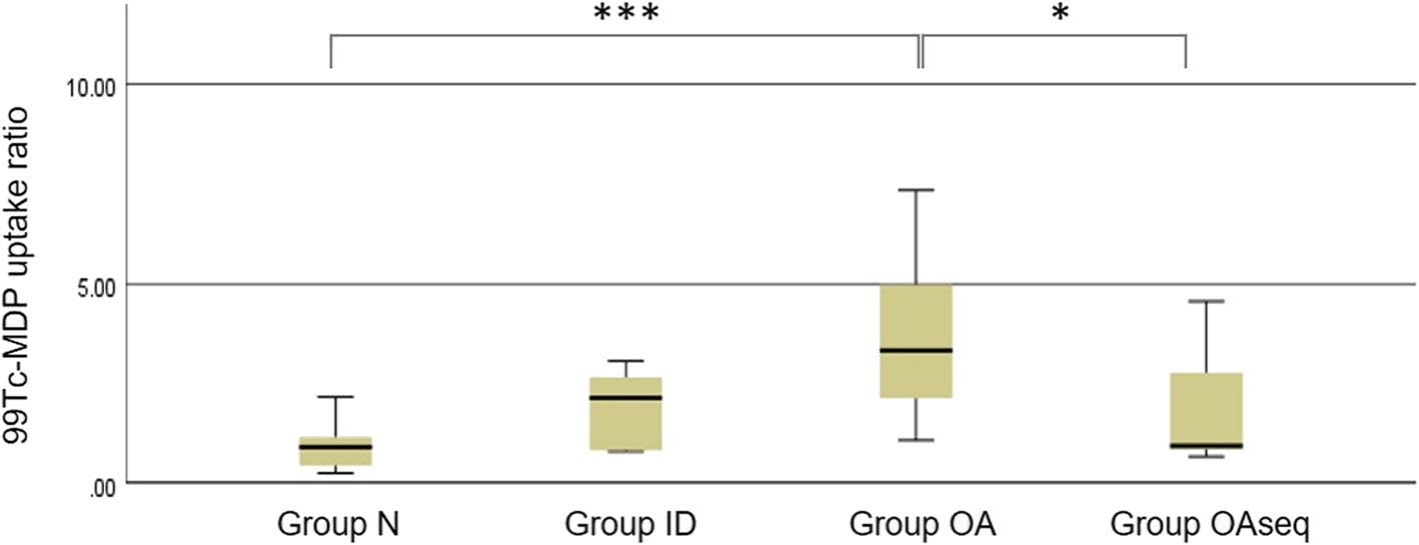
Diagram of the post hoc analysis. *p < 0.05; ***p < 0.001

Thirty-four TMJs of the 17 patients were divided into 4 groups according to the clinical and radiographic examinations of plain radiography (Supplemental Table). Twelve condyles were normal (group N), while 6, 9, and 7 condyles were diagnosed as internal derangement (group ID), osteoarthritis (group OA), and osteoarthritis sequelae (group OA_seq_), respectively. Group OA showed the highest uptake ratio, and group ID was the second and followed by group OA_seq_ and group N (Supplemental Table). Statistical significant difference of the ^99m^Tc-MDP uptake level among the 4 groups was observed (Supplemental Table and Fig. 3). Post hoc analysis demonstrated that statistical significances were attributed from the difference between group OA and group N (*p* = 0.000) and between group OA and group OA_seq_ (*p* = 0.048) (Fig. 3). This result could mean that fused SPECT/CT image can be effective in differential diagnosis of the osteoarthritis with osteoarthritis sequelae as well as normal state.

**Fig. 4:**
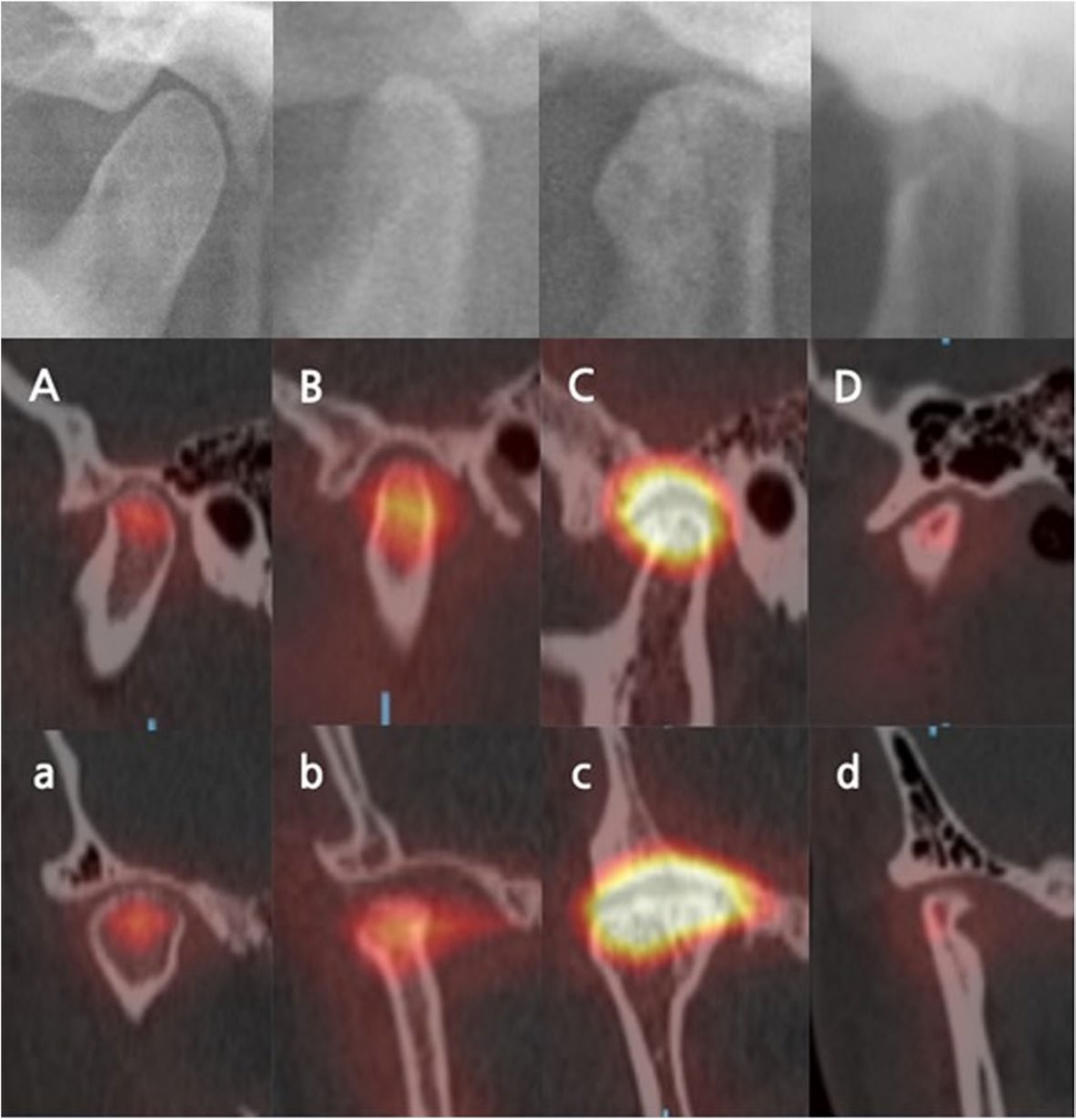
Fused images of bone SPECT and facial CT between groups. The scope of hot spot is greater in order of group OA, group ID, group OAseq, and group N. **A** Group N (normal), sagittal view. **a** Group N (normal), coronal view. **B **Group ID (internal derangement), sagittal view. **b** Group ID (internal derangement), coronal view. **C** Group OA (osteoarthritis), sagittal view. **c** Group OA (osteoarthritis), coronal view. **D** Group OA_seq_ (osteoarthritis sequelae), sagittal view. **d** Group OA_seq_ (osteoarthritis sequelae), coronal view

Additionally, comprehensive condylar image with hot spot was observed through the fused SPECT/CT images (Fig. 4). The scope of hot spot was greater in order of group OA, group ID, group OA_seq_, and group N, which was consistent with the order of uptake ratio.

### Group N (normal state)

Group N was defined as the condylar head without any abnormal symptom and feature on plain radiography. This group was the nature of control group. Twelve condyles were classified into the group N, and mean uptake ratio was 0.90, which was the lowest among the 4 groups (Supplemental Table and Fig. 3). Statistically significant difference between group N and group OA was observed in post hoc test (*p* = 0.000) (Fig. 3).

### Group ID (internal derangement)

Group ID was defined as an abnormal relationship between the articular disc and the mandibular condyle, articular fossa, and the articular eminence. This group represented the symptom of clicking sound during normal function, mouth opening limitation, temporary joint locking, and pain without any bony change. Six condyles belonged to this group. The mean uptake ratio in this group was 1.93, which was the second highest among the 4 groups (Supplemental Table and Fig. 3).

### Group OA (osteoarthritis)

In this group, bony change on condylar head such as bony erosion, joint space narrowing, sclerotic marginal deformity, subchondral low-density change, cortical thinning, cortical irregularity, subchondral sclerosis, subchondral cyst, cortical thinning, subchondral sclerosis, osteophyte, and irregularity was observed on plain radiography and CT image. Clinical symptoms such as mouth opening limitation, arthralgia, myalgia, clicking sound, and crepitus were also observed, which could mean the state of acute inflammatory osteoarthritis of TMJ. Nine condyles belonged to this group, and mean uptake ratio was 4.93 (Supplemental Table). This was the highest among the 4 groups, and statistical significances with group N (*p* = 0.000) and OA_seq_ (*p* = 0.048) were observed in post hoc analysis (Fig. 3).

### Group OA_seq_ (osteoarthritis sequelae)

In this group, similar bony change with group OA was observed on CT images. But this group showed no clinical symptoms except crepitus, which could mean termination of the TMJ inflammation. Seven condyles belonged to this group, and mean uptake ratio was 1.91. This was the third highest among the 4 groups (Supplemental Table and Fig. 3), and statistical significant difference with group OA was demonstrated (*p* = 0.048) (Fig. 3).

## Discussion

Osteoarthritis, degenerative change of the mandibular condyle, is largely caused by the excessive loads on TMJ, for example, masticatory movement of hard and tough foods, injury, and bad habits. If the excessive loads last, it may cause dislocation of temporomandibular disc, which in turn may cause pain during mouth opening, joint sound, and mouth opening limitation. If this progresses further, bony change on condylar head can be developed [1–5, 7]. At this time, clinicians can observe bony change on condylar head in the plain radiography. If such loads are reduced before advancing to severe degenerative change, symptoms disappear, and the affected condylar head by the inflammatory reaction can be healed through bone remodeling. However, it is difficult to judge that the observed bony change on condylar head is whether the inflammatory reaction is in progress or not. Therefore, it is necessary to differentiate whether it is bony change with inflammation status (osteoarthritis) or bony change without inflammation status (osteoarthritis sequelae) in TMD patients.

The objective of this retrospective study was to evaluate the clinical effectiveness of fused image of SPECT/CT for the evaluation of degenerative change of mandibular condylar head. This study performed a comparative analysis of ^99m^Tc-MDP uptake ratio obtained in the fused SPECT/CT image of the 4 groups, classified according to the findings from the plain radiography and clinical symptoms. Several applications of fused SPECT/CT image were performed previously. One research suggested the usefulness of fused SPECT/CT image which improves the sensitivity of the unilateral condylar hyperactivity [16]. In the mandibular growth assessment, the ^99m^Tc-MDP SPECT has provided the quantitative analysis where the increased uptake ratio on condylar head reflects the activity of the mandibular growth [17]. In osteonecrosis of the jaw patients treated with bisphosphonates, fused SPECT/CT image may be of value in increasing the diagnostic accuracy of bone scanning, providing a precise functional anatomic correlation for the definition of the extent of disease [18].

Accordingly, the used of fused SPECT/CT image was expected to be helpful to differentiate whether it is osteoarthritis or osteoarthritis sequelae in TMD patients. Increased ^99m^Tc-MDP uptake ratio was demonstrated in patients with internal derangement, osteoarthritis, and osteoarthritis sequelae than normal group (*p* = 0.003) (Supplemental Table and Fig. 3). This means that if clinical symptoms of TMJ appear, bone metabolism becomes more activated. Furthermore, there was a statistical significance between the group N and the group OA (*p* = 0.000) and between the group OA and group OA_seq_ (*p* = 0.048) (Fig. 3). The ^99m^Tc-MDP uptake ratio of the fused image could be effective to diagnose the stage of TMD. In addition, not only normal state but also osteoarthritis sequelae, which is often confusing in clinical situation, would be differentially diagnosed with the fused SPECT/CT image.

The result of this showed the possibility to distinguish whether it is in the stage of osteoarthritis (group OA) in the patients who show unclear bone resorption patterns on condylar head in a plain radiography or simply internal derangement (group ID). Appropriate clinically approach should be performed if detecting this before advancing to the severe degenerative change of condylar head. It is important to decide conservative approach or active joint movement therapy. If metabolic alteration on condylar head associated with acute inflammation was occurred, conservative approach should be preceded. Active joint movement therapy such as splint therapy or manipulation therapy would aggravate the degenerative change.

Limitation of this clinical study was not using of larger sample size that could have yielded more generalized results. Nevertheless, if the sample size was expanded, it is anticipated that statistical significance between the groups could be achieved. This would not only allow for the determination of bony changes on the condylar head with or without inflammation but also aid in identifying whether the TMJ is in the initial stage of osteoarthritis or simply experiencing internal derangement. Diagnosing certain TMJ conditions solely based on a patient’s subjective expressions of symptoms can be challenging and sometimes unclear. In such situations, fused SPECT/CT images present a valuable diagnostic tool. The integration of SPECT and CT scans provides comprehensive insights, aiding in the assessment of the osteoarthritis status of the TMJ. By combining these imaging techniques, clinicians can obtain a more accurate and objective evaluation of the condition, leading to improved diagnostic accuracy and better-informed treatment decisions.

## Conclusion

Fused SPECT/CT image can be an effective tool for evaluating degenerative changes in the mandibular condylar head. The technique demonstrated the ability to differentiate between normal TMJs and those with internal derangement, osteoarthritis, or osteoarthritis sequelae. This approach holds promise as a valuable method in clinical assessments of TMJ degeneration.

### Supplementary Information

**Additional file 1: Supplemental Table.** Groups according to the clinical and radiographic findings, and values and comparison of ^99m^Tc-MDP uptake ratio of the groups

## Data Availability

The datasets used and/or analyzed during the current study are available from the corresponding author on reasonable request.

## Abbreviations

SPECT: Single-photon emission computed tomography
CT: Computed tomography
^99m^Tc-MDP: Technetium-99 m methylene diphosphonate
Group N: Group normal
Group ID: Internal derangement
Group OA: Group osteoarthritis
Group OA_seq_: Osteoarthritis sequelae
TMJ: Temporomandibular joint
RDC/TMD: Research diagnostic criteria for temporomandibular disorder
ROI: Region of interest
