# Supplementary material for: The clinical effectiveness of fused image of single-photon emission CT and facial CT for the evaluation of degenerative change of mandibular condylar head
Source: Maxillofac Plast Reconstr Surg. 2023 Sep 27;45(1):33. doi: 10.1186/s40902-023-00399-1 (PMC10533429; doi:10.1186/s40902-023-00399-1)
Supplement: Supplementary file 1 — Additional file 1: Supplemental Table. Groups according to the clinical and radiographic findings, and values and comparison of 99mTc-MDP uptake ratio of the groups [file 40902_2023_399_MOESM1_ESM.docx]

Supplemental Table. Groups according to the clinical and radiographic findings, and values and comparison of ^99m^Tc-MDP uptake ratio of the groups

| No | Group | TMJ ID | Clinical findings | Plain Radiography | ^99m^Tc-MDP uptake ratio | Mean of ^99m^Tc-MDP uptake ratio (SD) | *P* value |
| --- | --- | --- | --- | --- | --- | --- | --- |
| 1 | Group N | 1L | occasional CS | No bony erosion | 0.73 | 0.90 (0.53) | 0.003** |
| 2 |  | 2R | occasional CS | No bony erosion | 0.37 |  |  |
| 3 |  | 3R | occasional CS | No bony erosion | 0.94 |  |  |
| 4 |  | 5R | occasional CS | No bony erosion | 0.37 |  |  |
| 5 |  | 6L | occasional CS | No bony erosion | 0.49 |  |  |
| 6 |  | 7L | occasional CS | No bony erosion | 2.16 |  |  |
| 7 |  | 8R | occasional CS | No bony erosion | 0.89 |  |  |
| 8 |  | 8L | occasional CS | No bony erosion | 0.25 |  |  |
| 9 |  | 9L | occasional CS | No bony erosion | 0.92 |  |  |
| 10 |  | 11L | occasional CS | No bony erosion | 1.34 |  |  |
| 11 |  | 13L | occasional CS | No bony erosion | 1.05 |  |  |
| 12 |  | 15L | occasional CS | No bony erosion | 1.26 |  |  |
| 13 | Group ID | 4R | CS, MOL, TJL | No bony erosion | 2.07 | 1.93 (0.94) |  |
| 14 |  | 4L | CS, TP | No bony erosion | 0.79 |  |  |
| 15 |  | 6R | CS, TP | No bony erosion | 0.82 |  |  |
| 16 |  | 10L | CS, TP, MOL, TJL | No bony erosion | 3.06 |  |  |
| 17 |  | 11R | CS, MOL | No bony erosion | 2.66 |  |  |
| 18 |  | 15R | CS, TP, MOL, TJL | No bony erosion | 2.20 |  |  |
| 19 | Group OA | 1R | CS, MOL, TJL, Arthralgia | Bony erosion, SMD | 4.71 | 4.93 (4.79) |  |
| 20 |  | 2L | CS, TJL, Myalgia | Bony erosion, SLDC, CT | 1.07 |  |  |
| 21 |  | 3L | CS, MOL, TJL, Arthralgia | Bony erosion, CI, SS | 1.09 |  |  |
| 22 |  | 5L | CS, Arthralgia, Myalgia | Bony erosion, CT | 4.98 |  |  |
| 23 |  | 7R | MOL, TJL, Arthralgia, Myalgia | Bony erosion, SMD | 7.34 |  |  |
| 24 |  | 12R | CS, Arthralgia, Myalgia | Bony erosion, JSN, SMD, SC | 16.54 |  |  |
| 25 |  | 13R | CS, TJL, Arthralgia | Bony erosion, JSN. CI | 3.33 |  |  |
| 26 |  | 14R | CS, Arthralgia, Myalgia | Bony erosion, JSN. CI | 3.17 |  |  |
| 27 |  | 17L | MOL, TJL, Myalgia | Bony erosion, SMD | 2.12 |  |  |
| 28 | Group OA _seq_ | 9R | Crepitus | Bony erosion, SMD | 4.56 | 1.91 (1.50) |  |
| 29 |  | 10R | Crepitus | Bony erosion, JSN | 3.09 |  |  |
| 30 |  | 12L | Crepitus | Bony erosion, JSN, SMD | 2.46 |  |  |
| 31 |  | 14L | ns | Bony erosion, JSN, CI | 0.83 |  |  |
| 32 |  | 16R | ns | Bony erosion, JSN, CI | 0.93 |  |  |
| 33 |  | 16L | ns | Bony erosion, JSN, CI | 0.66 |  |  |
| 34 |  | 17R | Crepitus | Bony erosion, FMF, SMD | 0.86 |  |  |

R: right condyle; L: left condyle

CS: clicking sound; MOL: mouth opening limitation; TJL: temporary joint locking; TP: temporary pain; JSN: joint space narrowing; SMD: sclerotic marginal deformity; SLDC: subchondral low density change; CT: cortical thinning; CI: cortical irregularity; SS: subchondral sclerosis; SC: subchondral cyst; FMF: flattening of mandibular fossa

ns: not specific; SD: standard deviation

*P* value was calculated with Kruskal-Wallis test.

***P*<0.01
